# Supplementary material for: COZOID: contact zone identifier for visual analysis of protein-protein interactions
Source: BMC Bioinformatics. 2018 Apr 6;19:125. doi: 10.1186/s12859-018-2113-6 (PMC5889581; doi:10.1186/s12859-018-2113-6)
Supplement: Supplementary file 2 — Software build. Executable binary file of the software tool. (ZIP 118,784 kb) [file 12859_2018_2113_MOESM2_ESM.zip › cozoid/platform/docs/testng-6.5.1-javadoc.zip!/org/testng/xml/XmlPackage.html]

XmlPackage (TestNG)


---


|  |  |  |
| --- | --- | --- |
| **Overview** \| **Package** \| **Class** \| **Tree** \| **Deprecated** \| **Index** \| **Help** | |  |
| **PREV CLASS**   **NEXT CLASS** | **FRAMES**    **NO FRAMES**     **All Classes** |  |
| SUMMARY: NESTED \| FIELD \| CONSTR \| METHOD | DETAIL: FIELD \| CONSTR \| METHOD |  |


---


## org.testng.xml Class XmlPackage

```
java.lang.Object
  org.testng.xml.XmlPackage
```

**All Implemented Interfaces:**: java.io.Serializable

---

``` public class XmlPackage extends java.lang.Object implements java.io.Serializable ```

This class describes the tag  in testng.xml.

**Author:**
:   Cedric, Alexandru Popescu

**See Also:**: Serialized Form

---

**Constructor Summary**

`XmlPackage()`

`XmlPackage(java.lang.String name)`


| **Method Summary** | |
| --- | --- |
| `boolean` | `equals(java.lang.Object obj)` |
| `java.util.List<java.lang.String>` | `getExclude()` |
| `java.util.List<java.lang.String>` | `getInclude()` |
| `java.lang.String` | `getName()` |
| `java.util.List<XmlClass>` | `getXmlClasses()` |
| `int` | `hashCode()` |
| `void` | `setExclude(java.util.List<java.lang.String> exclude)` |
| `void` | `setInclude(java.util.List<java.lang.String> include)` |
| `void` | `setName(java.lang.String name)` |
| `java.lang.Object` | `toXml(java.lang.String indent)` |

**Methods inherited from class java.lang.Object**

`clone, finalize, getClass, notify, notifyAll, toString, wait, wait, wait`

**Constructor Detail**

### XmlPackage

```
public XmlPackage()
```

---


### XmlPackage

```
public XmlPackage(java.lang.String name)
```


**Method Detail**

### getExclude

```
public java.util.List<java.lang.String> getExclude()
```

:   **Returns:**: the exclude

---


### setExclude

```
public void setExclude(java.util.List<java.lang.String> exclude)
```

:   **Parameters:**: `exclude` - the exclude to set

---


### getInclude

```
public java.util.List<java.lang.String> getInclude()
```

:   **Returns:**: the include

---


### setInclude

```
public void setInclude(java.util.List<java.lang.String> include)
```

:   **Parameters:**: `include` - the include to set

---


### getName

```
public java.lang.String getName()
```

:   **Returns:**: the name

---


### setName

```
public void setName(java.lang.String name)
```

:   **Parameters:**: `name` - the name to set

---


### getXmlClasses

```
public java.util.List<XmlClass> getXmlClasses()
```

---


### toXml

```
public java.lang.Object toXml(java.lang.String indent)
```

---


### hashCode

```
public int hashCode()
```

:   **Overrides:**: `hashCode` in class `java.lang.Object`

---


### equals

```
public boolean equals(java.lang.Object obj)
```

:   **Overrides:**: `equals` in class `java.lang.Object`


---


|  |  |  |
| --- | --- | --- |
| **Overview** \| **Package** \| **Class** \| **Tree** \| **Deprecated** \| **Index** \| **Help** | |  |
| **PREV CLASS**   **NEXT CLASS** | **FRAMES**    **NO FRAMES**     **All Classes** |  |
| SUMMARY: NESTED \| FIELD \| CONSTR \| METHOD | DETAIL: FIELD \| CONSTR \| METHOD |  |


---
